# Implications of Clonal Hematopoiesis in Hematological and Non-Hematological Disorders

**DOI:** 10.3390/cancers16234118

**Published:** 2024-12-09

**Authors:** Qi Zhang, Rita Yim, Paul Lee, Lynn Chin, Vivian Li, Harinder Gill

**Affiliations:** Department of Medicine, School of Clinical Medicine, LKS Faculty of Medicine, The University of Hong Kong, Hong Kong, China; kiki1226@connect.hku.hk (Q.Z.); ritayim@hku.hk (R.Y.); pl85@hku.hk (P.L.); lynnchin@hku.hk (L.C.); u3009010@connect.hku.hk (V.L.)

**Keywords:** clonal hematopoiesis, clonal hematopoiesis of indeterminate potential, clonal cytopenia of undetermined significance, myelodysplastic neoplasm, acute myeloid leukemia

## Abstract

Clonal hematopoiesis (CH) is associated with an increased risk of developing myeloid neoplasms (MNs) such as myelodysplastic neoplasm (MDS) and acute myeloid leukemia (AML). In general, CH comprises clonal hematopoiesis of indeterminate potential (CHIP) and clonal cytopenia of undetermined significance (CCUS). It is an age-related phenomenon characterized by the presence of somatic mutations in hematopoietic stem cells (HSCs) and hematopoietic stem and progenitor cells (HSPCs) that acquire a fitness advantage under selection pressure. Individuals with CHIP have an absolute risk of 0.5–1.0% per year for progressing to MDS or AML. Inflammation, smoking, cytotoxic therapy, and radiation can promote the process of clonal expansion and leukemic transformation. Of note, exposure to chemotherapy or radiation for patients with solid tumors or lymphomas can increase the risk of therapy-related MN. Beyond hematological malignancies, CH also serves as an independent risk factor for heart disease, stroke, chronic obstructive pulmonary disease, and chronic kidney disease. Prognostic models such as the CH risk score and MN-prediction models can provide a framework for risk stratification and clinical management of CHIP/CCUS and identify high-risk individuals who may benefit from close surveillance. For CH or related disorders, therapeutic strategies targeting specific CH-associated mutations and specific selection pressure may have a potential role in the future.

## Simple Summary

Clonal hematopoiesis (CH) predicts the development of myeloid neoplasms and other hematological malignancies. CH is also implicated as a risk factor for adverse outcomes in cardio-metabolic conditions. Future management of CH should target the somatic mutations associated with CH and the selection pressure that provide an advantage to hematopoietic stem and progenitor cells (HSCPs) that harbor significant CH-related somatic mutations.

## 1. Introduction

Clonal hematopoiesis (CH) is defined as a precursor state of myeloid neoplasm (MN). A number of studies have demonstrated that CH was associated with an increased risk of developing myelodysplastic syndrome (MDS) and acute myeloid leukemia (AML) [1,2]. Generally, CH includes CH of indeterminate potential (CHIP) and clonal cytopenia of undetermined significance (CCUS) [1]. CHIP is characterized by the presence of age-related somatic mutations with a variant allele frequency (VAF) of ≥2% and the absence of the history of cytopenia or any hematological malignancies [1]. Similarly, CCUS involves the presence of somatic mutations with VAF ≥ 2% and persistent (≥4 months) cytopenia in one or more peripheral blood cell lineages, with the absence of dysplastic features seen in MDS and not fulfilling the diagnostic criteria for other MNs [1]. CCUS has a high rate of progression to MN within 5 years, whereas CHIP may remain unchanged for a prolonged period. CH can be detected in various age groups, but it is more common among the elderly. Moreover, aging is suggested to be the strongest risk factor for CH as the hematopoietic capacity of hematopoietic stem cells (HSCs) declines gradually during aging [1,3]. The clone size of CH does not correlate significantly with age in the range of 60 to 110 years old. However, in the advanced age group, the number of CH-related mutations is strongly associated with progression to AML [4,5]. In general, individuals with CH are related to an absolute risk of 0.5–1.0% per year for MN, notably MDS and AML, and this risk is about 4 to 15 times higher than that for healthy individuals without CH [6]. CH is also an independent risk factor for cardiovascular diseases (CVDs) and all-cause mortality [7,8].

Age-related single nucleotide substitution, especially C-to-T substitution, is the most common CH mutation [1]. It is generally accepted that CH originates from the CD34^+^Lin^−^CD38^−^ HSCs [9]. CH-associated mutations may occur in genes that confer a selective fitness advantage and accumulate in HSCs and HS progenitor cells (HSPCs) with age, as an adaptive response. While aged HSCs and HSPCs are incapable of correcting all errors, a healthy individual aged 70 may acquire 350,000 to 1,400,000 mutations, and most of the reported CH mutations are also known mutations in frank diseases. These mutations can drive the process of clonal expansion and promote the subsequent preleukemic or leukemic transformation, thereby altering the function of terminally differentiated blood cells. In addition, CH mutations have also been detected in non-myeloid neoplasms and non-hematological disorders, signifying the necessity to elucidate the relationship between CH, CHIP/CCUS, and different diseases.

## 2. Myeloid Neoplasms

**Figure 1:**
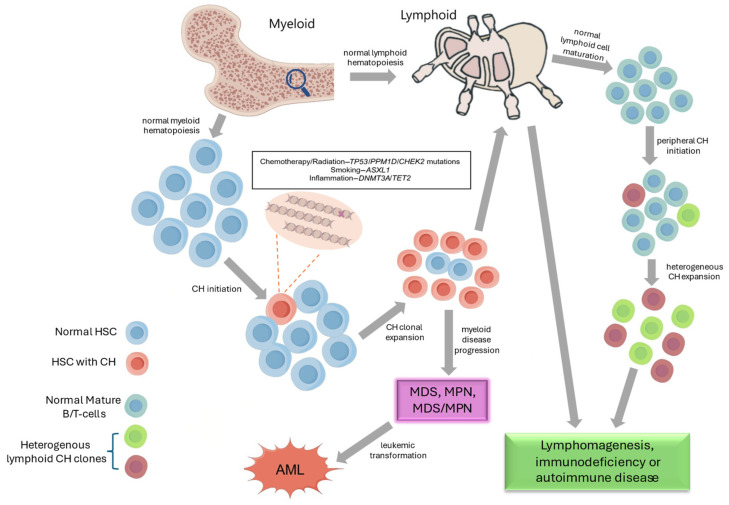
Progression and implications of clonal hematopoiesis. CH is initiated by the presence of driver mutations in the hematopoietic stem cells (HSCs). Those CH-related HSCs acquire a fitness advantage under selective pressures such as therapies like chemotherapy/radiation, smoking, and inflammation, which are risk factors that can promote the process of clonal expansion and malignant transformation. CH is associated with an increased risk of myeloid neoplasms. MDS: myelodysplastic neoplasm; MPN: myeloproliferative neoplasm; AML: acute myeloid leukemia.

PARP1-dependent microhomology-mediated end-joining (MMEJ) double-stranded break repair can cause mutations in genes like *ASXL1* and *SRSF2* at the initiation stage of CH [10]. Chronic inflammation in association with chemotherapy, radiation, or other specific environmental stimuli can promote accumulation of driver mutations somatically acquired in an age-dependent manner. The chemotherapy agents that typically play a role in such a process are cisplatin, etoposide, and doxorubicin. In addition to prolonged exposure to chemotherapy or radiation, the association of CH with unhealthy lifestyle behaviors is also reported. For instance, *ASXL1* mutations are strongly associated with smoking [11]. HSCs harboring CHIP-related somatic mutations will acquire a fitness advantage under the selection pressures leading to clonal expansion and myeloid transformation (Figure 1). Resistance to inflammatory signals can also enhance the clonal fitness of the mutant clones [12,13]. In the study by Jaiswal et al., approximately 4% of individuals with CH developed a hematological malignancy after an 8-year follow-up [14]. In patients with primacy malignancy, there was a 10-fold increase in the risk of therapy-related MN (t-MN). For those CH patients treated with cytotoxic agents, the median time of developing t-MN was further shortened to 2 years [15,16]. Upon autologous stem cell transplantation (ASCT), the risk of developing t-MN in patients with CH was as high as 14.1%, significantly higher than those without CH, whose incidence rate was 4.3% [17].

### 2.1. Implications of Clonal Hematopoiesis in Myelodysplastic Neoplasm and Acute Myeloid Leukemia

The risk factors for progression to MN in CH-related individuals usually include mutation of specific genes, clone size, and cooperative mutations. Epigenetic regulators, in particular *DNMT3A*, *TET2*, and *ASXL1*, also known as DTA, are the three genes most frequently mutated in CH [18]. Among these three genes, *DNMT3A* and *TET2* are involved in DNA methylation, and *ASXL1* regulates histone modification. Various mouse models have demonstrated that *DNMT3A* mutations could promote aberrant inflammation by activating the inflammasome complex and inducing pro-inflammatory T-cell polarization. According to Jeong et al., knocking out *DNMT3A* endowed the long-term HSCs with a strong competitive advantage and skewed the hematopoiesis towards the granulocytic lineage [19]. The process of myeloid transformation in murine HSCs with *DNMT3A*R878H mutation could be driven by the mutation of the nucleophosmin 1 (*NPM1*) gene. *DNMT3A*-mutant clones had earlier clonal outgrowth at a young age and showed decelerated clonal expansion in a natural aging process; this makes *TET2* mutation the most prevalent CH driver in the elderly [20]. Moreover, *TET2*-mutant CHIP/CCUS also showed the strongest association with CVD [21].

In contrast to the functional implication of *TET2* in CH being well studied, the implication of *ASXL1* mutations in CH is not well characterized. In fact, *ASXL1* mutations in CH are commonly frameshift variants clustered at the last exon, and these frameshift INDEL are considerably variable across individuals [22]. The expression model of truncated *ASXL1* by Nagase et al. showed that such hotspot mutations could perturb normal hematopoiesis and assert a positive selection pressure to accumulate additional driver mutations [23]. According to Avagyan et al., the ability of the *ASXL1*-mutant clone to establish clonal dominance could be abrogated by the biallelic loss of *NR4A1*, which is an immunomodulator [12]. Nevertheless, the high variability of the frameshift variants increased the difficulty in studying the functional impact of *ASXL1* mutations.

In addition to DTA, mutations of DNA damage-repairing genes *PPM1D* and *TP53* are also crucial in CH pathogenesis, and mutations of these two genes can be frequent in t-MN. Prior exposure to cytotoxic agents was shown to cause loss-of-function mutations of *PPM1D* and *TP53*, and loss of *TP53* could trigger CH in HSCs under constant radiation stress [24]. Other epigenetic regulators like *IDH1* and *IDH2* were also closely associated with CH despite occurring at a lower frequency than DTA mutations. An analyzed cohort of 292 patients showed that among the common *IDH2* mutations, mutation at R140, R132, or R172 was closely associated with CH and AML [25]. In MDS/AML-related CH, spliceosome components (*SF3B1*/*SRSF2*/*U2AF1*) can be frequently mutated as early mutations in AML and MDS derived from CHIP or CCUS [26]. However, these events are rare in pediatric diseases. It is possible that a fully functional spliceosome plays a significant role in the establishment of a hematopoietic system, notably in fetal and neonatal stages. Along with the development of normal hematopoiesis, the mutated splicing factor clones can remain at a low level until the acquisition of cooperation mutations and subsequent progression to leukemia [27].

Among different types of gene mutation, either as cooperative or standalone mutations, signaling pathway genes (e.g., *JAK2*, *CBL*, *GNB1*, and *GNAS*) have been reported occasionally as low-frequency CH. For *JAK2*, *JAK2*V617F is the most common mutation that is implicated in Philadelphia-negative myeloproliferative neoplasms (MPNs), and this covers polycythemia vera (PV), essential thrombocythemia (ET), and primary myelofibrosis (PMF). *JAK2*V617F is mostly found in younger individuals, and it was associated with increased risk of thrombosis and, more importantly, increased risk, and hence incidence, of CVD [21]. Although the allele burden of this mutation in CH was always very low (ranging from 0.06% to 1.73%), it was associated with high levels of IL-6 and IL-18 production and chronic inflammation [28].

Recently, Bernstein et al. analyzed somatic mutations in whole blood samples from 200,618 individuals in the UK Biobank. Besides the common CH-associated mutations, they identified 17 additional mutated genes, namely, *ZBTB33*, *ZNF318*, *ZNF234*, *SPRED2*, *SH2B3*, *SRCAP*, *SIK3*, *SRSF1*, *CHEK2*, *CCDC115*, *CCL22*, *BAX*, *YLPM1*, *MYD88*, *MTA2*, *MAGEC3*, and *IGLL5.* Clones with these mutations expanded in variant frequencies and clone sizes in an age-dependent manner, comparable to classical CH drivers. Inclusion of these novel fitness-inferred genes in the selection criteria has increased the detection rate of CH (restricted to large clones > 0.1) by 18% in the UK Biobank cohort [29]. When Zeventer et al. longitudinally studied cytosis and cytopenia of 3359 individuals, a large heterogeneity of the evolutionary landscape was revealed. Despite being heterogenous, mutations of *JAK2* and spliceosome genes (*SF3B1*/*SRSF2*/*U2AF1*) showed the highest growth, whereas *DNMT3A* and *TP53* mutated clones showed only a marginal increase in the clonal expansion rate over time. When focusing on the MNs, mutations of *TP53*, *JAK2*, spliceosome, and *KRAS*/*NRAS* genes conferred the highest risk for MN. While the observed clonal expansion was independent from general cancer-promoting factors (e.g., smoking) or cytosis/cytopenia, this indicated the strong predictive power of somatic mutations [26].

### 2.2. Clonal Hematopoiesis in Chronic Myeloid Leukemia, BCR::ABL1+

Although somatic mutations are commonly detected in several MNs, systematic investigation of CH in chronic myeloid leukemia (CML) is considerably limited. In a serial study in which preleukemic CH clones were detected in 37% of CML patients, CH acquisition, persistency, and clearance were associated with a variety of clinical outcomes. Regardless of successful treatment responses from tyrosine kinase inhibitors (TKIs), CML patients could show a persistent mutation burden. As expected, patients who acquired secondary somatic mutations during TKI treatment finally failed the therapy. On the other hand, patients exhibiting mutation clearance were correlated with mixed clinical outcomes [30]. In Branford et al.’s study, CH was detected at the time of cessation (TOC) of treatment, and these CH^TOC^ values were characterized by ≥1 CH clone with VAF ≥ 2.0% or multiple clones in which the combined VAF was ≥2.0%. The presence of CH^TOC^ was an independent predictor of treatment-free remission (TFR) at 12 and 84 months. These CH^TOC^ events impacted TFR if the CH clones were able to outcompete the driver *BCR*::*ABL1* clone. In a minority of patients with delayed relapse, competition between the CH clone and the leukemic clone was suspected during the first 12 months upon TKI cessation [31].

### 2.3. Clonal Hematopoiesis in Classical Philadelphia Chromosome-Negative Myeloproliferative Neoplasm

Mutations in *JAK2*, *CALR*, and *MPL* are major disease drivers for myeloproliferative neoplasm (MPNs), and can initiate or promote MPNs with or without other co-mutations. Nevertheless, it is the process of clonal expansion rather than the acquisition of these driver gene mutations that plays a pivotal role in progression to MPNs. *JAK2*V617F mutation occurs in more than 95% of PV patients and about half of the ET or PMF patients [32]. In the context of *JAK2* mutation in CH, the time required for CH transformation into MPN was 5 to 15 years [20,33]. A VAF of >2% or a continuously increased VAF during follow-up time could be associated with a high rate of conversion from CH to MPN [34,35]. The frequency of *JAK2*V617F CH is 15-fold higher than that of *CALR* CH, but *CALR* mutation is linked to a higher rate of clonal expansion, therefore shortening the conversion time from gene acquisition to CH mutation and then MPN diseases [36].

For *JAK2*V617F, which is associated with high levels of cytokine production and chronic inflammation, it is not surprising that the proinflammatory environment also functions as a factor that promotes CH to MPN [37]. Notably, IL-1β secreted by *JAK2*V617F mutant cells can favor early clonal expansion and promote the CH transformation into MPNs. Impairment of HSC clonal expansion and reduced mesenchymal stromal cells resembling the CH-like phenotype were reported in mouse HSCs with *JAK2*V617F mutation while *IL-1β* was knocked out [38]. Knockout of *IL-1β* also suppressed the engraftment efficiency in secondary transplantation models and reduced subsequent MPN initiation in comparison with donor HSCs with only *JAK2*V617F [38]. In MPN patients with homozygous *IL-1β* polymorphisms having an elevated IL-1β production, it is not surprising to see treatment of anemic patients with CH mutations by anti-IL-1β antibody showed favorable hemoglobin responses [38]. Supported by other reports on chronic or systemic inflammations associated with secondary solid tumors, the therapeutic role of modulating the inflammation in *JAK2*V617F CH individuals should be investigated [39,40].

### 2.4. CIN, ICUS, CCUS, and MDS

The MDS Study Groups introduced the term idiopathic cytopenia of undetermined significance (ICUS) in 2005. ICUS refers to cytopenia(s) of more than 4 months and it neither fulfills the minimal diagnostic criteria of MDS nor shows any evidence of underlying disease associated with cytopenia [41,42]. Isolated neutropenia among patients with ICUS is known as ICUS-N or chronic idiopathic neutropenia (CIN). The frequency of detecting CCUS in patients with ICUS ranges from 38% to 64% in different studies [43,44,45]. In comparison with nonclonal ICUS, CCUS is clinically more important, as individuals with CCUS are strongly associated with a higher probability of progression to MN [44,46,47]. In this context, Tsaknakis et al. studied the mutation profile and clinical significance of gene mutations in 185 CIN patients. In particular, 11.35% of the patients harbored 25 mutations across six genes with a median VAF of 12.75% [48]. This rate of mutations was significantly lower than the previously reported CCUS mutation frequency of at least 38% in ICUS patients, indicating that CIN could be a distinct ICUS subgroup [43,44,45]. In this study, the most prominent gene mutations were *DNMT3A*, *TET2*, *IDH1*/*2*, *SRSF2*, and *ZRSR2*. With the VAF of these mutant clones in MN-transformed patients being at least >10%, the genes most strongly related to MN were *SRSF2* and *IDH1* [49].

In contrast to pancytopenia being less prevalent, isolated cytopenia was significantly more common in CCUS than in MDS [44,50,51]. Conventional discrimination of CCUS from MDS is reliant on cytomorphologic examination of BM. Nevertheless, accuracy of this morphologic/microscopic approach is often limited due to sub-optimal sample quality and subjective interpretation by pathologists [50]. The progression risk from CCUS to MDS was highly variable and correlated with multiple factors based on different studies. It includes, but is not limited to, specific genes, clone sizes, and numbers of mutations [44,50,52,53,54,55]. Huber et al. analyzed 222 CCUS cases and 698 MDS cases, and a total of 351 mutations in 28 CH genes were detected in the CCUS cohort [56]. In comparison with MDS, CCUS patients were observed with fewer CH mutations per patient, which was in concordance with another previous study [50]. In the context of the mutation profile, CCUS patients frequently carried DTA mutations with the corresponding prevalences of individual genes being 32%, 28%, and 14% respectively. In addition, mutations of *DNMT3A* and *PPM1D* were even more prevalent in CCUS than in MDS. Huber et al. also demonstrated a comparable mutational spectrum between CCUS and MDS. These major differences in the frequency and VAF of mutant genes indicated the existence of different subgroups within CCUS [56].

In the context of large mutations, chromosomal abnormalities can initiate disease progression of CCUS to MNs independently from other parameters [54]. Cytogenetic alterations could be found in 27% of CCUS and 43% of MDS patients. Moreover, loss of chromosome Y was more frequent in CCUS than in MDS, while deletion of chromosome 5q is more common in MDS [56]. While the mosaic loss of chromosome Y is aged-related, there are controversies about whether these genetic lesions are proper risk factors for hematological malignancies of different lineages. It is debatable if small mutations are more inclined to MNs, whereas chromosome Y mosaicism is predisposed to lymphoid neoplasms [57,58].

## 3. Non-Myeloid Hematological Disorders

### 3.1. Lymphoma and Autologous Stem Cell Transplantation

Previous studies revealed an association between CH in the peripheral blood stem cell (PBSC) harvest and t-MN in non-Hodgkin lymphoma (NHL) patients after ASCT. In a population-based study from Husby et al., 25.5% of NHL patients were detected with at least one CH mutation. Confounded by the fact that study cohort with CH were biased towards aged individuals, CH in PBSC was not strongly linked to inferior overall survival (OS) [59]. This was in concordance with Gibson et al. and Husby et al., who further reported in patients exposed to high-dose pre-transplant chemotherapies that mutations of DNA repair genes (*PPM1D*, *TP53*, *RAD21*, and *BRCC3*) were frequent. These mutations conferred an inferior OS, thereby making them powerful as independent predictors. For those patients who had higher rates of developing t-MNs, the therapeutic preponderance of successful eradication of the CH clones at the time of transplantation could be indispensable [17,59].

Similar to NHL, the association between CH in the PBSC product and risk of developing t-MN in HL patients also remains to be clarified. In a recent retrospective study of 321 HL patients, CH-associated gene mutation was detected in 14.3% of these patients who underwent ASCT. The most prominent mutations included *DNMT3A*, *TET2*, *PPM1D*, and *TP53* [60]. With the presence of CH in the PBSC product being an independent risk factor of t-MN, the genes with the strongest negative predictability were *TP53* followed by *PPM1D.* Prior to cytotoxic treatments, the median OS in patients with a CH-associated DNA repair mutation was significantly shorter than in those without any mutations (2.2 years vs. 9.0 years). In addition, the 8-year cumulative incidence of CH-associated t-MN was 7.4% in patients with NHL, and this was much lower than that in HL patients (18.5%) [60]. However, this association between the PBSC product and risk of t-MN and other disease-associated mortality could not support a consensus, as there were contradictory observations from other studies in both HL and NHL [15,17,59].

### 3.2. Aplastic Anemia

T-cells from the adaptive immune system are the progeny of HSCs. Clonal T-cell expansions from CH are common during clonal maturation in the lymphoid tissue (Figure 1). These CH-related mutations may alter the number and function of non-leukemic T-cells or even disrupt the immune system. Moreover, inflammation may directly affect the proliferation and self-renewal of HSCs, thereby altering the CH process [61]. An extreme case is aplastic anemia (AA), in which cytotoxic T-cell expansions are involved in the disease pathogenesis, and specific CH genotypes appear to acquire a competitive advantage in the process of autoreactive HSC destruction mediated by cytotoxic T-cells [62,63]. For instance, HSCs with *DNMT3A* or *ASXL1* mutations could evade cytotoxic T-cells [64]. In CD8^+^ T-cells, mutations could be frequently observed in the JAK-STAT and MAPK pathways, which confer the clonality of CD8^+^ T-cells. Of these, *PIGA*, *BCOR*, or *BCORL1* mutations could be frequently yet exclusively detected in AA patients, and these mutations tend to disappear or exhibit stable clone sizes [63].

In CD3^−^ cells (T-cell depleted fraction), mutations of *DNMT3A*, *BCOR*/*BCORL1*, and *ASXL1* were frequent but the transcriptomic pattern was not exclusive to CD3^−^ cells. At the DNA level, in patients with *BCOR/BCORL1*, mutations were detected in the CD3^+^ cells (T-cell enriched fraction) but the CD3^−^ fraction was only positive for *ASXL1* or *DNMT3A* mutations [63]. Clinical data were consistent with this inference that a better response to immunosuppressive therapy could be found in AA patients with mutation of *PIGA*, *BCOR*, or *BCORL1*. As expected, these patients were less likely to develop leukemic transformation in comparison with those patients with *DNMT3A* or *ASXL1* mutations [64].

## 4. Implications of Clonal Hematopoiesis in Cellular Therapy and Cytotoxic Therapy

### 4.1. Allogeneic Hematopoietic Stem Cell Transplantation

In allo-HSCT, donor HSCs have CH mutations and could exist but evade detection by rigorous health and genetic screening. The engrafted clones can expand and undergo clonal evolution without the introduction of hematological malignancy before allo-HSCT. However, undesirable complications are still sometimes inevitable when the donor CH clone is pathogenic. For instance, unexplained anemia can be observed in patients receiving donor cells with a *DNMT3A* mutation [65]. In the study by Gibson et al., 85% of the donor clones, including small clones with VAF < 1%, persisted throughout the long-term engraftment [66]. In severe conditions, donor cell leukemia (DCL) has been reported; this is a rare but serious complication affecting 0.1% of all recipients [67,68,69,70]. DCL can be initiated from the proliferative stress, which is originally essential for hematopoietic re-establishment in the bone marrow environment, and thereby successful engraftment. Moreover, the immunosuppressive regimen upon allo-HSCT could impose a negative effect by evading immunological surveillance, eventually promoting leukemic transformations.

Similar to the mutation profiles of HL or NHL receiving ASCT as mentioned above, *DNMT3A* or *TET2* mutations in donors were common but they hardly triggered DCL. The most potent gene that had higher importance than these two genes was, in fact, *TP53* in donor cells, which could easily drive DCL. Second to somatic *TP53* mutation, germline *DDX41* or spliceosome mutations (*SF3B1*/*SRSF2*/*U2AF1*) were also risk factors for DCL, and a combinatorial effect from these three mutations was postulated [66]. For the more common but less potent donor-related *DNMT3A* mutation, recipients infused with *DNMT3A* mutated donor CH in fact benefited from better survival due to reduced relapses. However, this beneficial effect from *DNMT3A* mutations was confined to recipients receiving the conventional calcineurin-based therapy as prophylaxis for graft-versus-host disease (GvHD). At least partially attributed to inflammatory cytokine, recipients transplanted with *DNMT3A* mutated donor cells had a higher plasmatic level of IL-12. This enhanced IL-12 secretion was positively correlated with IL-1β, IL-4, IL-5, and IFN-γ. On the other hand, it was negatively correlated with IL-8, IL-10, IL-22, and TNF-α. Moreover, such augmentation of the pro-inflammatory cytokine network in recipients, at least in part, contributed to the chronic GvHD (cGvHD) observed in those patients [66].

With the tactful balance between desirable graft-versus-leukemia and tolerable GvHD being pivotal for a successful HSCT, the clonal dynamics of donor CH in this aspect has been studied by Frick et al. and Gibson et al. [66,71]. Interestingly, donor CH had no effect on thrombocyte engraftment time, but it was associated with a subtle improvement in leukocyte engraftment time. Among the many CH mutations in donors being reported, *DNMT3A* mutations were associated with a higher incidence of cGvHD. The risks of non-relapse mortality and cumulative incidence of relapse/progression (CIR/P) were also lower in these patients transplanted with donor cells harboring *DNMT3A* mutations [66]. Frick et al. further indicated that recipients without achieving complete remission (CR) prior to transplantation could benefit from donor-engrafted CH as this could prevent relapse potentially involving cGvHD [71].

### 4.2. Chimeric Antigen Receptor T-Cell Therapy

In patients undergoing CD19 or B-cell maturation antigen (BCMA) chimeric antigen receptor (CAR) T-cell therapy, toxicities like cytokine release syndrome (CRS) in relation to inflammation after CAR-T infusion is the major complication. Approximately 24% of patients who received CAR-T carried at least one CH, with *DNMT3A* and *CHEK2* being the two most common genes mutated. These patients with CH had a significantly higher risk of developing grade ≥ 2 CRS, compared to those without CH (60% vs. 28%). This association remained significant even when the patient with putative germline *SH2B3* was excluded from the analysis, confirming the poor outcome was due to somatic events. However, except for CRS, CH showed no correlation with other common clinical outcomes like progression-free survival or OS. There was also no correlation with immune effector cell-associated neurotoxicity syndrome in CAR-T therapy [72].

In Kapadia et al.’s study, which longitudinally monitored CH in lymphoma patients receiving CD19 CAR-T cells, CH was detected in 54%. Those CH carriers tended to have a better disease-free survival, while there was no significant association between the occurrence of CH with OS, inflammatory toxicities, and most of the common clinical parameters. Remarkably, most patients were found to have multiple CH clones, and *DNMT3A* was the most frequently mutated gene followed by *PPM1D* and *CHEK2* [73]. All these three genes were reported by other studies focused on cell-based therapies, but myeloid recovery post-CAR-T therapy was not influenced by these mutations [72]. Kapadia et al. also observed a higher level of CAR-T cell persistence in CH carriers compared with non-carriers when the infusion dose was the highest, suggesting the beneficial effect from CH was dose-dependent. However, they could not determine the best expansion levels of CAR-T benchmarked by the CAR-T cells’ persistency along time [73]. In the context of clonal dynamics, longitudinal tracking of a single clone in recipients revealed that small clones expanded shortly after CAR-T product infusion, and they expanded more efficiently than large CH clones. In the infused T-cell product, matched mutant clones, which confirmed the donor origin of the CH clone detected in the recipient, were also present at a low magnitude, but this presence did not affect the cell product’s immunophenotype or transduction efficiency [73].

### 4.3. Cytotoxic Therapy

Anticancer therapies like conventional chemotherapy and radiotherapy are risk factors for CH to trigger complications, as these regimens may place the cancer survivors at an elevated risk for t-MN. Constant exposure to alkylating agents and bleomycin was significantly associated with high risk of developing therapy-related CH, and hence secondary cancers. Meanwhile, prior exposure to new agents such as immune checkpoint inhibitors was not strongly linked to this undesirable condition [74,75,76,77]. Although therapy-related CH was not uncommon (~25%), 90% of cases remain clonally stable for decades without developing t-MNs [78]. Some exceptional cases with known pathogenicity include mutations of *TP53*, *PPM1D*, or *CHEK2*, and they are closely related to t-MN. For the impact of *PPM1D* in therapy-related AML and MDS, mutation of such often led to truncation of the PPM1D protein, which can no longer suppress p53, thereby evading cell death during chemotherapy [75,79].

Despite the fact CH is well-known to be associated with advanced age, the incidence rate of CH in pediatric cancer patients is unexpectedly high. By comparing the telomere attrition rate with the community control group, the rate of underlying HSC division in CH carriers was found to be no different from normal [74]. Among different types of hematological malignancies and solid tumors, several types of tumors were more prone to develop therapy-related CH. These include HL, NHL, and ALL for hematological malignancies and soft-tissue sarcoma, germ cell tumor, rhabdomyosarcoma, and neuroblastoma for solid tumors [74]. Among the common anti-cancer therapeutics, alkylating agents, followed by radiotherapy then bleomycin, were more likely to introduce therapy-related CH. Interestingly, therapy-related CH was less likely to occur in cancer survivors receiving radiotherapy compared with other non-radiation-based regimens [74]. In the context of molecular characteristics of therapy-related CH, mutation of *STAT3* and *TP53* was reported. Hagiwara et al. discovered *STAT3* mutation to be a novel variant specific to therapy-related CH, especially to HL, and such mutations were significantly enriched in CD8^+^ cells. While age-related CH showed that larger clone size could be a predictor of clonal expansion, the size of the therapy-related CH clone estimated by VAF failed to serve as a good predictor for subsequent clonal expansion hence failed to predict disease transformation [74].

## 5. Clonal Hematopoiesis and Non-Hematological Disorders

**Figure 2:**
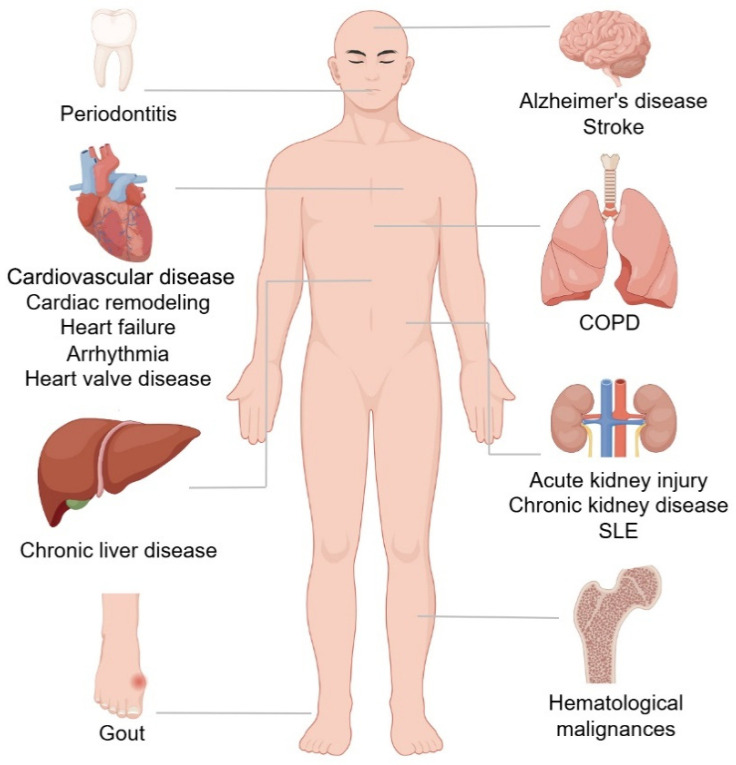
Malignant and nonmalignant outcomes of CHIP. CHIP carriers have an increased risk of many malignant and nonmalignant disorders. The progression of hematological malignances cardiovascular disease, COPD, and chronic liver disease is mostly related to CHIP, but CHIP carriers are associated with a decreased risk of Alzheimer’s disease.

Despite epidemiologic studies showing CH carriers have an elevated risk of hematologic malignancies, the actual risk is only 0.5–1% per annum [13]. CHs have been reported in a wide range of organ systems and different diseases, with cardiovascular disease being the most documented condition (Figure 2). Therefore, it is inadequate to conclude that hematological malignancies are responsible for all CH-associated deaths when the total risk of mortality is as high as 40%, significantly higher than the risk of transformation into any kind of hematologic malignancy [14]. In fact, atherosclerotic cardiovascular events (CVEs) account for a high proportion of CH-associated mortality, especially in individuals with a high VAF CH clone [80]. In most cardiovascular studies of CH, DTA mutations were shown to increase the risk of incident coronary heart disease (CHD) and ischemic stroke. This, in reality, accounted for approximately 40% of the mortality in CH carriers, and CH-associated mutations were more likely to develop CHD when the VAF was 10% or higher [14,21,81].

### 5.1. Clonal Hematopoiesis in Cardiovascular Diseases

#### 5.1.1. Coronary Artery Disease, Cardiac Remodeling, and Heart Failure

A recent study of the Chinese population consisting of 6181 participants supported the correlation between CH and coronary heart disease (CHD). In this Chinese study cohort, the risk of CHD was 1.42-fold higher among the 1100 CH carriers, a considerably higher risk than that in the study by Schuermans et al. comprised of a mainly Black/African American and Hispanic/Latinx population [82,83]. When harboring *TET2*-CH mutations with VAFs larger than 10%, a substantially elevated risk of CHD was exhibited [82]. For 532 individuals who were small-clone carriers (VAF 0.5–2%), there was a 1.33-fold higher risk of CHD compared to those without CH. This indicated that CHD risk attributed to CH was not negligible even for small clones [82]. When studying the functional role of these CH mutations in cardiovascular disease (CVD), *TET2* and *DNMT3A* mouse models were created using CRISPR by Sano et al. [84]. Both *TET2* and *DNMT3A* deletions in HSCs resulted in increased angiotensin II-mediated cardiac hypertrophy, reduced cardiac function, and worsened cardiac fibrosis [84]. In Schuermans et al.’s study of 8090 participants, for those with any CH-associated mutation, they had a 28% increase in risk of heart failure with preserved ejection fraction (HFpEF) [83]. Of note, *TET2* mutation was associated with a 2.4-fold higher risk of HFpEF, and it was considered an independent risk factor for HFpEF. In contrast, HF with reduced ejection fraction was not associated with any particular CH [83].

When *TET2* was more potent than *DNMT3A*, disruption of both genes promoted the inflammatory responses. Interestingly, the inflammation patterns were not identical between the two models. In particular, *IL-6* and *CCL5* expression were upregulated in both conditions but the upregulation of *IL-1β* was not as consistent between the two CRISPR models [84]. In another similar study by Sano et al., their chronic heart failure model showed that *TET2* depletion imposed a negative impact on cardiac remodeling and function, and this was likewise mediated by elevated IL-1β [85]. Similar to Lin et al., treatment with an *NLRP3* inhibitor reduced secretion of IL-1β in *TET2*-deficient macrophages and ameliorated atherosclerosis or heart failure caused by *TET2*-depleted myeloid cells [85,86].

In addition to NLRP3-mediated inflammation, the AIM2 inflammasome also played a role in atherosclerosis in relation to CH. Fidler et al. discovered that macrophage-restricted *JAK2*V617F could induce DNA replication stress and activate the AIM2 inflammasome, worsening the process of atherosclerosis [87]. The mutant macrophages also showed increased expression of proliferative markers that could alter the plaque stability in vivo¸ yet this undesirable inflammation was reversible by treatment with the JAK2 inhibitor Ruxolitinib [87]. Collectively, these results indicated a causal relationship between inflammasome activation of macrophages with CH-associated mutations during the pathogenesis of CVD. This further signifies the therapeutic potential by early intervention or elimination of the CH clones.

#### 5.1.2. Cardiac Arrhythmia

Although CH is associated with CHD and HF, the pathological role of CH in cardiac arrhythmias is not as well understood. In this context, Lin et al. proposed a modest association between *TET2* CH and atrial fibrillation (AF) in the UK population. In both atherogenic and non-atherogenic mouse models, inactivated *TET2* in the HSC population enhanced the risk of AF. The elevated risk was attributed to increased expression of NLRP3 inflammasomes and dysregulated cytosolic deposition of calcium within cardiomyocytes. In vitro modulation of proinflammatory cytokines IL-1β and IL-6 could rescue inflammation mediated by increased expression of *NLRP3* [86]. In another study of a significantly larger UK population, Saadatagah et al. confirmed the association between increased risk of AF with large CH clones of *TET2* and *ASXL1* (but not *DNMT3A*). *TET2* and *ASXL1* mutant clones were further found to be associated with a high level of IL-6 and multiple echocardiographic indices for cardiac remodeling [88]. In the subsequent study by Schuermans et al., CH was suggested to confer an increased risk of supraventricular arrhythmia, ventricular arrhythmia, and bradyarrhythmia, and this association was stronger with large CH clones in comparison with small clones [89]. In addition to their earlier study showing that *TET2*-CH was specific to HFpEF, they subsequently discovered *TET2* and *TP53* were CH specific to cardiac arrest, and *PPM1D* was CH specific to AF [89].

#### 5.1.3. Valvular Heart Diseases

Chronic inflammation and advanced age are not surprisingly high-risk factors for severe aortic valve (AV) stenosis, and previous studies have shown the role of CH in different heart diseases. In order to characterize this in greater depth, Mas-Peiro et al. performed a deep sequencing study on *DNMT3A and TET2* in 287 patients with severe AV stenosis and after transfemoral aortic valve implantation (TAVI) [90]. Although patients with and without mutation of *DNMT3A* and *TET2* showed comparable outcomes in most parameters, patients carrying *DNMT3A* and *TET2*-CH had a significantly higher mortality even though TAVI was successful. Downstream cytometric analysis further detected pro-inflammatory T-cell polarization in *DNMT3A*-CH carriers with an elevated Th17/Treg ratio. A similar pro-inflammatory feature in *TET2*-CH carriers was also observed, as evidenced by increased circulating non-classical monocytes (CD14^dim^CD16^+^), which are known to secrete high levels of pro-inflammatory cytokines [90].

#### 5.1.4. Cerebrovascular Disease

Qiu et al. analyzed the genomic profiles of 6016 Chinese patients with acute ischemic stroke, of which 3.7% were identified with the presence of CH, and the most common mutations were *DNMT3A* (30%) and *TET2* (11.4%). CH carriers were associated with recurrent stroke, ischemic stroke, and combined vascular events [91]. Similar frequencies were also reported in a recent meta-analysis by Sigh et al., which showed that DTA mutations and *JAK2*V617F were the most frequent form of CH. However, a large heterogeneity was observed across different study cohorts, with clone sizes measured by VAF being the major variability contributing to the deviations [92].

### 5.2. Chronic Obstructive Pulmonary Disease

In addition to age, chronic obstructive pulmonary disease (COPD) is associated with cumulative exposure to cigarette smoke, and this is regarded as one of the major environmental risks in COPD and other respiratory diseases. A retrospective analysis was performed to investigate the genomic/exomic profiles of 48,835 patients from multiple cohorts. This included the UK Biobank and the COPDGene cohort; the latter was the largest COPD study in the Trans-Omics for Precision Medicine program [93]. With the DTA mutations being the most common CH, as expected, CH was one of the determining factors for developing COPD and advanced stages of COPD in most of the cohorts. Both *DNMT3A* and *TET2* mutations led to a significantly higher risk of advanced COPD, but the statistical impact of *DNMT3A* was comparatively limited compared to *TET2*. There was also a significant association between CH and COPD in individuals with increasing magnitudes of smoking history [93]. In addition, CH carriers also had restricted pulmonary function, and this was more prominent in moderate or severe COPD patients. In animal models, mice lacking *TET2* in hematopoietic cells also had accelerated development of emphysema and inflammation benchmarked by pulmonary inflammation. Assisted by single-cell sequencing technology, the IFN-γ signaling was found to be activated upon *TET2* inactivation, and this inflammatory signature involved not only T-cells but also NK cells being the predominant source of IFN-γ [93].

### 5.3. Chronic Liver Diseases

Chronic liver disease progresses from steatosis to inflammation and fibrosis [2]. Recently, Wong et al. investigated the association between CH and chronic liver disease in 214,563 individuals from four different cohorts. CH was associated with a two-fold increase in the risk of prevalence and incidence of chronic liver disease, which is significantly higher in comparison with individuals without CH [94]. Mouse models transplanted with *TET2*-deficient HSCs also showed more severe liver inflammation and fibrosis. These processes were mediated by the NLRP3-inflammasome and elevated levels of IL-6, CXCL1, CCL22, and CCL17, which are downstream inflammatory cytokines released by *TET2*-deficient macrophages [94]. In fact, the association between CH and chronic liver disease was regarded as genotype-dependent. In particular, *TET2*-mutant individuals were related to a 5.4-fold higher risk of chronic liver disease, while *JAK2*-mutant individuals were associated with a 17.6-fold increase in risk [94].

### 5.4. Autoimmune and Musculoskeletal Disorders

#### 5.4.1. Systemic Lupus Erythematosus (SLE) and Rheumatoid Arthritis (RA)

Savola et al. investigated associations between CH and the clinical phenotype in RA, and discovered CH in 17% of RA cases [95]. *DNMT3A* and *TET2* were the most frequent mutant genes, in agreement with the mutational spectrum in the healthy control. Notably, *DNMT3A* R882H or other substitutions at the same amino acid position, which were classical pathogenic mutations in MDS/AML, were not observed in RA patients [14,18,95,96]. With the mutation hotspot in the RA patients being different from MDS or AML, these CH clones in RA were likewise very stable for decades [97]. In another study that focused on SLE, David et al. studied the association between CH and the predisposition to CVEs in SLE patients. Out of the 438 SLE cases studied, a total of 63 somatic mutations were identified in 47 patients. In both SLE and RA, CH were likely to be monogenic events, and 62.5% of the CH mutations detected in SLE were *DNMT3A* mutations [98]. The prevalence of CH was associated with the age of SLE diagnosis and CH did not impact the incidence of CVEs. Although vascular disease was the major cause of morbidity and mortality in SLE, the association between CH and adverse outcomes in SLE needs further evaluation [98].

#### 5.4.2. Gout

For mild immunological conditions involving CH like gout, *TET2*-mutant CH was detected as an increased risk factor for gout from exomic analysis of 177,824 individuals from the MGB Biobank and UK Biobank [99]. There is a modest correlation of CH with gout, as the prevalence of gout was significantly different across the two cohorts, with a higher risk of gout only observed in the MGB Biobank cohort, but not the UK Biobank cohort. For cases with CH clones having a 10% VAF or greater, the risk could be increased by 28% [99]. In mouse models transplanted with *TET2*-knockout HSCs, injection of monosodium urate crystal into mouse paws exacerbated *NLRP3*-dependent IL-1β secretion. This proinflammatory status resulted in edema formation and macrophage-predominant inflammatory infiltrates. In contrast, treatment of these transgenic mice with an NLRP3 inhibitor could suppress NLRP3-dependent secretion of IL-1β in *TET2*-knockout mice, thereby ameliorating the mice from the inflammation [99].

### 5.5. Renal Diseases

In nephrology, the association between CH and acute kidney injury (AKI) was reported by Vlasschaert et al. in multiple large cohorts [100]. In conjunction with AKI, the association of CH with chronic kidney disease (CKD) was also confirmed by screening 172 patients from two independent cohorts with advanced CKD. In both studies by Vlasschaert et al., patients with AKI or CKD were mostly detected as having DTA mutations [100,101]. In CKD, CH was associated with decreased hemoglobin, increased use of erythropoiesis-stimulating agents, and elevated parathyroid hormone levels. Risk of kidney failure within 5 years was also found to be 2.2-fold greater in patients with CH [102]. When focusing on AKI, individuals with AKI commonly harbored *TET2* and *JAK2* mutations, and the clone sizes were often large. Interestingly, *DNMT3A* was in fact the most common CH, but *DNMT3A* CH was not associated with AKI. In those patients that carried non-*DNMT3A* CH, the AKI pattern was twice as hard to resolve in these CH carriers compared with those without CH mutations [100]. With *TET2* being the most common CH with a pathological impact on AKI, mouse models were used to study the association between CH and AKI severity. In mice transplanted with CD45^+^ cells deficient in *TET2*, exaggerated AKI patterns were observed followed by ischemia reperfusion injuries as a result of *TET2*^-/-^ cell dominance in the intrinsic myeloid kidney cell population. The pathogenic role of *TET2* in AKI was associated with post-AKI kidney fibrosis and greater renal proinflammatory macrophage and neutrophil infiltration [100].

### 5.6. Periodontitis

Unlike the AKI and CKD, in which *DNMT3A* is the least implicated gene among the DTA mutations, the functional role of *DNMT3A* in dental diseases is better understood. CH from *DNMT3A* mutation can result in elevated osteoclast precursors in BM and osteoclastogenic macrophages in the periphery. Hui Wang et al. retrospectively discovered *DNMT3A*-driven CH with an increased prevalence of periodontitis among 4946 community adults from the Atherosclerosis Risk in Communities cohort. The functional role of *DNMT3A* R878H in periodontitis was also confirmed using mice models. Upon engraftment of CD45^+^ BM cells carrying *DNMT3A* R878H, recipient mice developed periodontal inflammation and bone loss. The impaired regulatory T-cell immunosuppressive activity, which is IL-17-dependent inflammation, activated downstream neutrophil responses and, eventually, periodontitis and periodontal bone loss. These inflammatory responses could be suppressed by treatment with the mTOR inhibitor rapamycin, and this was marked by effectively suppressing the clonal expansion of CD45.2 leukocytes positive for *DNMT3A* R878H [103].

### 5.7. Alzheimer’s Disease

Microglia, the macrophage-like hematopoietic cell in the brain, plays an important role in the pathogenesis of Alzheimer’s disease (AD). In a meta-analysis of 1362 AD patients and 4368 individuals without AD, the risk of AD was lower by 36% in patients with CH-associated mutations. This protection from AD neuropathologic change and dementia was confirmed by subsequent Mendelian randomization analysis [104]. In AD patients who also underwent brain autopsy, the mutations detected in blood specimens were also detected in the brain-resident microglia from CH carriers. By measuring the regional β-amyloid deposits, the presence of CH was associated with better AD neuropathologic statuses, and this protection from CH was also observed using a separate staging system [104]. While the *APOE* genotype is the strongest genetic risk factor for AD, different genotypes of *APOE* confer different AD characteristics, and hence risks [105]. When the CH mutations in the AD patients were studied based on different *APOE* genotypes, there was a consistent trend that CH carriers in each *APOE* stratified group showed better scoring and staging [104].

## 6. Prognostic Models and Clinical Management for Clonal Hematopoiesis

In the field of CH, the clinical impact of clones with VAF below 2% is less comprehensively investigated. Nearly all middle-aged adults carry very low but stable CH mutations (VAF < 1%). Similar to two other recent studies, Husby et al. also concluded that low-level clones were of less predictive value [59]. In fact, most CHIP/CCUS patients are identified by incidental findings of routine check-ups and counseling of other diseases, or even found in the process of screening for healthy donors of recipients with hematological malignances. It is accepted that routine screening for CHIP/CCUS in the general population during regular hospital appointments may not be currently recommended. Since CHIP/CCUS is highly prevalent in the general population, the provision of a simple prognostic framework for known CHIP/CCUS may aid CH research and clinical management. Using the UK Biobank as a training dataset, the CH risk score (CHRS) is created as a prognostic model and it is the first validated tool created for this purpose. It can distinguish patients with a high MN risk from most CHIP/CCUS individuals [106]. In brief, the prognostic variables of CHRS include single mutation in *DNMT3A*; presence of multiple mutations; VAF ≥ 20%; high-risk mutations (splicing factors *SRSF2*, *SF3B1* and *ZRSR2*, *JAK2*, *IDH1*, *IDH2*, *RUNX1*, *FLT3*, or *TP53*); mean corpuscular volume ≥ 100 femtoliters; red cell distribution width ≥ 15%; cytopenia; and age ≥ 65 years. With these key variables in the model, the CHRS can classify patients into three major CHIP/CCUS risk groups (high-, intermediate-, and low-risk), and it can also demonstrate the absolute low risk of developing MN in the vast majority of CHIP/CCUS [106].

Similar to CHRS, MN-predict is also available to predict future risk of disease progression to AML, MDS, or MPN. It combines serum chemistry, body mass index, and molecular features to generate a time-dependent prediction [107]. Both CHRS and MN-predict are tools available for risk stratification of CHIP/CCUS and can provide a framework for management strategies of CH patients in hematology clinics. All CCUS patients need BM aspiration/biopsy and cytogenetic analysis, and they may also need to repeat NGS (from PB) when facing new clinical symptoms. CHIP/CCUS patients at low or intermediate risk can be followed up annually with complete blood count (CBC) monitoring, and bone marrow examination with cytogenetic and molecular studies can be repeated if there are significant hematological changes. For high-risk CHIP/CCUS patients, more frequent monitoring of CBC (e.g., every 3–6 months) may be required, and reassessment of bone marrow with cytogenetic and molecular studies is performed if there are significant changes in the hematological parameters. Nevertheless, no definite criteria are available to determine which patients with CHIP/CCUS will benefit from early therapeutic intervention [108,109].

## 7. Potential Therapeutic Strategies for Clonal Hematopoiesis

To date, there is no established treatment for CH. Various studies have explored effective treatment strategies for specific mutations. Among DTA mutations, *TET2* shows the best potential as a therapeutic target despite *TET2* not being the among the three most-prevalent genes. In animal models, treating *TET2*-deficient mice with high-dose vitamin C could mimic *TET2* restoration and reverse the aberrant proliferation and renewal of HSPCs. This treatment strategy is also effective in treating leukemic cells positive for the driver *FLT3* mutation [110]. In the clinical setting, the therapeutic effect of vitamin C was previously demonstrated by two studies focusing on MN patients with or without vitamin C deficiency [111]. In the light of these trials, the efficacy investigation of vitamin C supplementation has been extended to CCUS or high-risk MN patients. In this ongoing clinical trial (ClinicalTrials.gov identifier: NCT03682029), 55 patients were randomized into either placebo or vitamin C-treatment groups. While data on the primary endpoint and other correlatives are pending completion, there was a prolonged OS in the vitamin C-treated group compared to the placebo, and the frequency of serious adverse events was reduced from 43% to 27% [112].

Rapamycin treatment plays a significant role in the process of suppressing inflammation, which involves the suppression of IL-17-dependent inflammation and neutrophil responses involved in CH-related periodontitis [103]. In addition, inhibitors targetingIL-6 and TGFβ signaling-induced gene expression could be drugs with good potential in ameliorating an age-related lineage bias [113]. In the phase 3 CANTOS randomized trial, Woo et al. examined the efficacy of the anti-IL-1β neutralizing antibody (canakinumab) on solid tumors and CH mutations. Among the 10,061 patients with a history of myocardial infarction and increased pro-inflammatory markers, 3923 patients were profiled molecularly. The TET2-mutated patients treated with canakinumab had the lowest incidence of solid tumors [114]. With *TET2* being very common as a CH-associated mutation in cardiovascular diseases, canakinumab likely plays a positive role in preventing cancers and confers the clinical benefits in disease management.

## 8. Conclusions

CH can be considered as a collection of various clonal abnormalities; many subtypes are etiologically linked to adverse malignant or nonmalignant outcomes. Genomic data from high-throughput sequencing technology have provided a new approach of exploring patients diagnosed with CHIP/CCUS. Fortunately, both CHRS and MN prediction models can provide an intuitive and adoptable framework for risk stratification. The access to publicly available molecular and clinical data enables continuous discoveries for better risk stratification of CH. This will eventually assist the development of prospective therapeutic interventions for CHIP/CCUS-related disorders.
